# Optomechanical Analysis of Gait in Patients with Ankylosing Spondylitis

**DOI:** 10.3390/s25061797

**Published:** 2025-03-14

**Authors:** Vedran Brnić, Frane Grubišić, Simeon Grazio, Maja Mirković, Igor Gruić

**Affiliations:** 1 Department of Rheumatology, Physical and Rehabilitation Medicine, Sestre milosrdnice University Hospital Center, 10000 Zagreb, Croatia; frane.grubisic@kbcsm.hr (F.G.); simeon.grazio@kbcsm.hr (S.G.); 2 School of Medicine, University of Zagreb, 10000 Zagreb, Croatia; 3 Faculty of Kinesiology, University of Zagreb, 10000 Zagreb, Croatia; 4 Kinematika—Polyclinic for Orthopedics, Physical Medicine and Rehabilitation, 10000 Zagreb, Croatia; maja.mirkovic@kinematika.hr; 5 Faculty of Electrical Engineering and Computing, University of Zagreb, 10000 Zagreb, Croatia

**Keywords:** ankylosing spondylitis, gait analysis, kinematics, kinetics, pedobarography, Kinect v2

## Abstract

Ankylosing spondylitis (AS) is a chronic inflammatory rheumatic disease associated with alterations in posture and gait. The aim of this study was to assess the gait of AS patients using pedobarography and a markerless motion capture system. This is the first study of this population to combine these two methods. Twelve AS patients and twelve healthy controls were enrolled in this study. An instrumented gait analysis of both groups was performed using pedobarography and Microsoft Kinect v2. The AS group was significantly older than the controls (*p* < 0.05). The AS group showed a significantly lower relative pressure distribution in the front-right quadrant (*p* = 0.01) and a significantly higher relative pressure distribution in the rear-right quadrant (*p* = 0.05) on the static pedobarography. The AS group also had a higher peak force in the midfoot on the dynamic pedobarography (*p* < 0.05). The AS group had a significantly shorter stride length (*p* = 0.01). No significant differences between the groups were found in their hip flexion/extension and adduction/abduction, knee flexion, or ankle dorsiflexion/plantarflexion angles. This study shows significant alterations in the pedobarographic and spatiotemporal, but not in the kinematic, gait parameters of AS patients. These alterations represent a feature of AS and not antalgic adjustments. Rehabilitation programs for AS patients could be tailored according to the results of an instrumented gait analysis and should include balance and gait exercises.

## 1. Introduction

Ankylosing spondylitis (AS) is a chronic inflammatory rheumatic disease and the most common form of spondyloarthritis. It predominantly affects the axial skeleton (sacroiliac joints and spine) and is characterized by inflammatory back pain and impaired spinal mobility. It can also be associated with peripheral arthritis, enthesitis, and extra-articular manifestations [1]. Disease onset is usually in the twenties, and it is more common in males (males–females ratio 3:1) [2,3]. An earlier age of onset is associated with worse functional outcomes [4]. The global prevalence of AS is 0.1% to 1.4% [5]. The disease is related to functional impairment, work disability, and decreased quality of life [5,6].

Syndesmophyte formation and ankylosing of the spine in AS patients lead to spinal rigidity and postural alterations: head protraction, increased thoracic kyphosis, and loss of lumbar lordosis [7]. This results in a forward and downward shift of the center of mass. Compensation for this postural shift is mostly achieved through increased knee flexion and ankle plantarflexion [8]. Due to hip arthritis, which is the most common peripheral manifestation of the disease, with a prevalence of 24–36% [2,9], hip flexion contracture often develops, limiting hip extension as another possible compensatory mechanism. Altered posture, along with back pain, decreased spinal mobility, and foot abnormalities, have been hypothesized to be the main reasons for gait alterations in AS patients [8,10,11]. Gait impairments can increase fatigue during ambulation in these patients [12,13] and are associated with a reduced quality of life [14]. However, considering gait as one of the primary functions of the musculoskeletal system and its importance for functional independence and quality of life, only a few studies have performed instrumented gait analyses of AS patients.

The methods for the quantitative assessment of motor functions, including gait, are valuable tools for planning specific rehabilitation interventions. An instrumented gait analysis can provide objective and quantifiable information on the spatiotemporal, kinematic, kinetic, and electromyographic parameters.

The spatiotemporal and kinematic parameters can be assessed with a three-dimensional (3D) gait analysis, electronic walkway, inertial sensors, and electrogoniometers. A kinematic analysis includes information on the position, speed, acceleration, and angles between body segments. The main types of modern motion capture systems used for spatiotemporal and kinematic gait analysis are marker-based stereophotogrammetry systems and markerless systems. 

The markerless technology used in this research was originally developed in 2010 by Microsoft, with its Kinect Xbox 360^®^, also known as Kinect v1, followed by Kinect v2 in 2014 and Azure Kinect in 2019. The main advantages of these systems over marker-based systems are their considerably lower price; the mobility of the systems, which can be used outside a laboratory environment; and the simplicity and speed of calibration, setup, and use. Although preliminary studies found that Kinect has good validity for the spatiotemporal parameters, but poor validity for the kinematic parameters [15], later studies have shown that the joint angles measured by Kinect follow the trend of traditional marker-based systems and confirmed the Kinect v2 as an acceptable tool for kinematic gait analysis, especially the hip and knee angles in the sagittal plane, with limitations for the measurement of ankle movements [16,17,18,19].

Kinetic gait parameters can be assessed with a classical ground reaction force dynamometer or pedobarography. In this study, we used pedobarography to evaluate the interactions between the foot and the supporting surface and to obtain the plantar force and pressure distribution data. The plantar pressure can be measured during postural activities (static pedobarography) and dynamic activities, like the gait (dynamic pedobarography) [20]. Pedobarography can provide information about postural control and serve as a good indirect indicator of postural dysfunction [21], an important characteristic of AS. It can indicate the presence of compensatory mechanisms of the hip, knee, and ankle joints through the effects of these compensations on the foot, the most distal segment of the kinetic chain.

According to a systematic review by Soulard et al. [22], five studies have used laboratory gait measurements to assess the gait of patients with AS [23,24,25,26,27]. The main findings of this review described the gait pattern of patients with AS as more cautious [23,25], less stable [24,25], and less variable [25] than the healthy controls. The AS patients also had decreased stride length [23,26], decreased lower-limb angles in the sagittal plane [23,25,26], and increased hip abduction and external rotation [26]. However, as the author noted, the results of this review should be taken with caution, as the sample sizes were small and comparing studies was difficult due to the different parameters and protocols used.

Only a few studies have analyzed the posture and gait of AS patients using pedobarography, with inconclusive results. In the static pedobarographic measurements by Mesci et al. [28], the ratio of the pressure distribution over the proximal half of the foot to the distal half of the foot was significantly greater in the AS patients compared to the healthy control group. The other two studies did not find differences in the static pressure distribution in AS patients compared to healthy controls [29,30]. Only one study used dynamic pedobarography, which showed increased dynamic forefoot and midfoot pressures in the AS patients compared to the healthy controls [29]. Further research is needed to improve the understanding of static and dynamic postural control mechanisms in AS patients.

The aim of this study was to assess the gait characteristics of AS patients using pedobarography and a markerless motion capture system. To our knowledge, no previous study has used a markerless motion capture system for a gait analysis of this population. Furthermore, combining pedobarographic and kinematic methods is a novel approach to gait analysis in AS patients. The practical intention and reasoning behind this experimental design and approach was to ‘fill the gap’ between the assessments of AS patients with ‘gold standard’, high-cost instrumentation (VICON, ELITE…) and the widespread, user-friendly tools for kinematic analysis (2D Kinovea or likewise, and 3D RGB-D systems, such as Azure Kinect, Stereolabs ZED, Intel RealSense, and others). Due to this gap, many clinicians do not use any instrumentation in clinical gait assessments.

## 2. Materials and Methods

### 2.1. Study Design

This study was designed as a cross-sectional study. It was conducted from August 2021 to April 2022 at the Department of Rheumatology, Physical and Rehabilitation Medicine of Sestre milosrdnice University Hospital Center in Zagreb, Croatia. Laboratory gait analysis was conducted at Kinematika—Polyclinic for Orthopedics, Physical Medicine and Rehabilitation, an accredited gait analysis laboratory collaborating with the Faculty of Kinesiology, University of Zagreb.

### 2.2. Participants

The sample size for this study was calculated using the difference in stride length between patients with AS and healthy controls in the study by Zebouni et al. [23]. With a significance level of 0.05 and power of 80%, the sample size was estimated at 12 for each group. G*Power for Windows v3.1.9.4. was used for sample size and effect size calculations [31].

Twenty-four participants were enrolled in this study, including 12 AS patients (AS group) and 12 healthy controls (HC group). AS patients were recruited from the patients treated at the department. All patients met the modified New York diagnostic criteria for AS [32], with a duration of the disease of at least 12 months at the time of enrollment. The control group consisted of healthy volunteers. The exclusion criteria for both groups were as follows: (1) neurological, cardiovascular, or metabolic diseases that could affect gait; (2) fracture of or orthopedic surgery on the spine, pelvis, or lower limbs; (3) scoliosis; (4) pain in the spine, pelvis, or lower limbs of more than 4/10 on the pain visual analog scale (VAS); (5) hip, knee, or ankle joint contracture; (6) use of walking aid; (7) uncorrected visual or auditory impairment; and (8) cognitive disorder.

### 2.3. Data Collection

#### 2.3.1. Demographic and Anthropometric Data

Demographic data (sex and age) and anthropometric measurements (height and weight) of all participants were obtained. Joint contracture of the hips, knees, and ankles was ruled out by clinical examination. All participants were instructed to take analgesics before gait analysis in case of severe pain, and the pain level in the spine, pelvis, and lower limbs should not have been more than 4/10 on the VAS, which was checked for all participants prior to gait analysis.

#### 2.3.2. Gait Analysis

The instrumented gait analysis of all participants was performed using pedobarography and a markerless motion capture system. The evaluations of the AS patients were carried out at least four hours after they woke up, to minimize the effect of morning stiffness. All participants wore thin black socks and close-fitting, but comfortable, black clothing that did not impede lower limb motion. This was required to minimize the influence of socks on pedobarographic measurements and the influence of clothing on the accuracy of Kinect’s tracking.

#### Instrumentation and Setup

For pedobarographic measurements, a 0.5 m Entry Level Footscan^®^ (Materialise, Leuven, Belgium) pressure distribution platform was used. The dimensions (length × width × height) of the platform were 578 mm × 418 mm × 12 mm, and the active sensor area of the platform measured 488 mm × 325 mm. It consisted of 4096 resistive sensors arranged in a 64 × 64 matrix, with a data acquisition frequency of 300 Hz and a pressure range of 1–127 N/cm^2^. The platform was embedded in the middle of a walkway that measured 372 × 100 cm. The platform was connected to a computer via USB 2.0 using the supplied cable.

Simultaneously with pedobarographic measurements, kinematic gait data were acquired using a markerless motion capture system, Microsoft Kinect^®^ v2 (Microsoft Corporation, Seattle, WA, USA). The Microsoft Kinect v2 sensor comprised an RGB (Red, Green, Blue) camera, an infrared camera, and three infrared projectors. The sensor utilized the time of flight principle for depth estimation. The RGB camera had a resolution of 1920 × 1080 pixels, and the infrared camera had a resolution of 512 × 424 pixels. The acquisition rate of both cameras was 30 Hz. The depth range was 0.5–4.5 m, and the depth field of view was 70.6° × 60° (horizontal × vertical). Minimal system requirements for Kinect v2 were as follows: 64-bit (x64) processor, physical dual-core 3.1 GHz (2 logical cores per physical) or faster processor, dedicated USB 3.0 controller, 4 GB RAM, a graphics card that supports DirectX 11, and a Microsoft Kinect v2 sensor.

**Figure 1:**
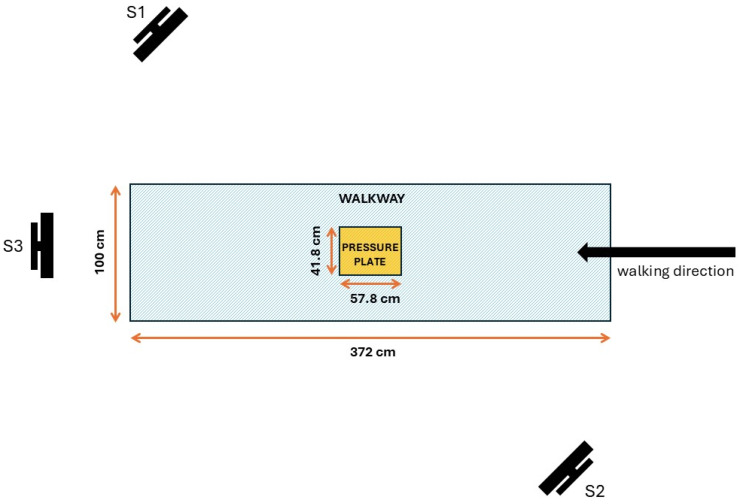
Overview of pedobarography and multi-Kinect v2 setup.

To reduce the self-occlusion of the tracked subject and to ensure more accurate tracking, three Kinect v2 sensors were used in this study, as per study by Geerse et al. [33]. Each sensor was connected to a separate computer via USB 3.0 using the supplied cables. Sensors were positioned at a height of 80 cm. The first sensor (S1) was placed on the right side of the walkway, with an orientation of 45° to the walking direction, 2.5 m from the center of the pressure platform. The second sensor (S2) was placed on the left side of the walkway, directly opposite the first sensor, with an orientation of 45° in the reverse direction of walking, 2.5 m from the center of the pressure platform. The third sensor (S3) was placed 4.3 m from the start of the walkway, facing the participant when walking (Figure 1). Synchronous start of measurement of all sensors was attained with the application Microsoft Garage Mouse without Borders [34]. It is a product that allows for control of up to four computers with a single mouse and keyboard.

#### Protocol for Gait Analysis

A standardized protocol for pedobarography was designed according to recommendations by Giacomozzi [35] and adjusted for the length of the walkway. The weight calibration of the Footscan system was performed before each measurement session according to the manufacturer’s manual. Participants were then instructed to walk at a self-selected, moderate speed along the walkway in one direction, without looking at their feet or aiming for the platform. At the end of the walkway, they returned to the starting position by walking freely beside the walkway, which was not included in the analysis. Before data collection, all participants completed a few practice trials along the walkway. During practice trials, images generated by each Kinect sensor were checked to ensure that the participant remained within the depth range and field of view throughout the trial. During practice trials, each participant also determined a suitable starting position, usually two or three steps before contact with the platform, depending on their individual step length. This ensured normal walking speed at the point of contact with the platform. One step on the platform was recorded per trial, and three steps on the platform with each foot were recorded per participant. Trials that resulted in incomplete footprint or trials with obvious adjustments in gait pattern upon contact with the platform were repeated.

After this, the dynamic measurement of the first step in gait initiation was carried out. Each participant determined a suitable starting position, a step away from the platform, based on individual step lengths obtained during practice trials. Then, each participant initiated their normal gait from the starting position, with the first step landing on the platform. One step on the platform with each foot was recorded per participant.

For the static measurements, participants were instructed to stand upright, with both feet on the platform, for five seconds, as still as possible, facing forward, arms at their sides, and toes directed forward with their self-selected distance between the feet.

### 2.4. Data Analysis

#### 2.4.1. Pedobarography

Pedobarographic data were processed by the Footscan^®^ Essentials v9.10.4 software package (Materialise, Leuven, Belgium). Pedobarographic measurement was visually represented as a pedobarogram, a two-dimensional spatial map with similar pressure distribution areas represented with correlating colors. The color scale varied from blue (minimum pressure) to red (maximum pressure). The length and width of both feet of all participants were calculated from the software data. The software automatically generated a graphic representation of ten anatomical zones of the foot. Numerical pedobarographic values for these zones are available from a more advanced software package, which was not available at the time, so each pedobarogram was manually divided into three anatomical regions: forefoot, midfoot, and heel. For each pedobarogram, a matrix with pressure values corresponding to each sensor was overlayed with a semi-transparent colored pedobarogram comprising software-generated corresponding anatomical zones. Before this, colored pedobarograms were imported into Microsoft Excel (Microsoft Corporation, Seattle, WA, USA) as .jpg files and adjusted to the size of the pressure matrix.

Selected outputs from dynamic pedobarography (first step with the right foot and first step with the left foot) and static pedobarography (standing position) were analyzed.

In dynamic pedobarographic measurements, peak pressures were measured and calculated for each participant in each foot region, followed by the calculation of peak forces. Peak pressure (N/cm^2^) was the highest pressure value registered by each sensor during the measurement. Peak force (“max F”, in Newtons) was the highest force value registered for the whole foot and for three foot regions (forefoot, midfoot, and heel) separately.

**Figure 2:**
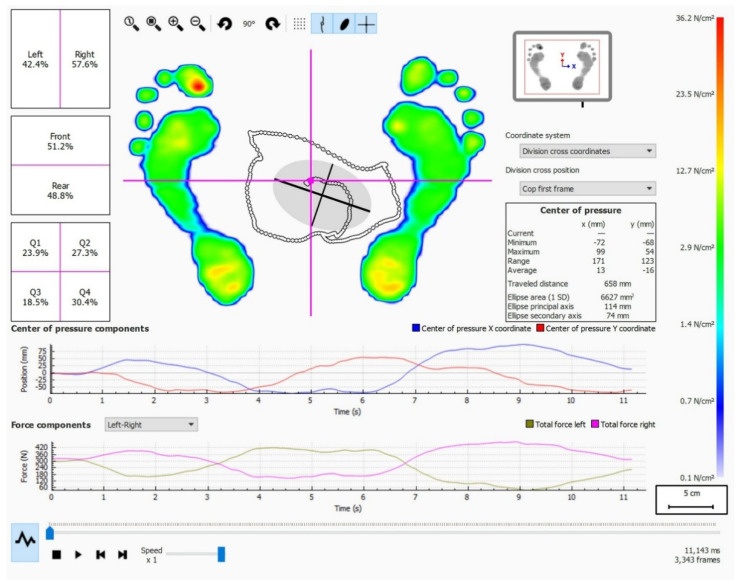
Static pedobarographic measurements—relative pressure loads (%) across quadrants (default Balance Analysis screen in the Footscan manual). Reproduced with permission from RSscan International/Materialise, Installation Guide and User Manual footscan^®^ System with footscan^®^ 9, published by RSscan International, 2017 [36].

Static measurements were analyzed using the Balance Analysis feature of Footscan software. It evaluated the pressure distribution and the displacement of the center of pressure (Figure 2). Measurements were divided into four quadrants (Q1, Q2, Q3, and Q4). The intersection of these quadrants indicated the center of pressure of the measurement. Measurements were expressed in percentages and indicated how the force was spread across each quadrant over the measured time.

#### 2.4.2. Motion Capture System

For broadcasting Kinect data on a computer, we used Kinectron, an open-source peer server for Kinect v2 and Kinect Azure that makes real-time motion capture data available on a browser through an application programming interface [37]. After installing Kinect for Windows Software Development Kit 2.0 and the accompanying drivers, Kinectron software was installed.

**Figure 3:**
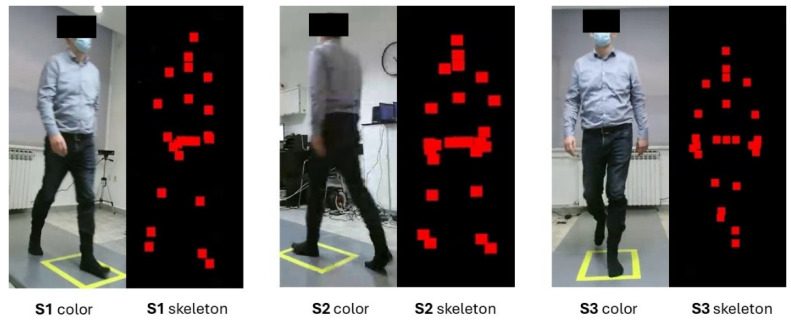
Kinect v2 “Color” and “Skeleton” images.

**Figure 4:**
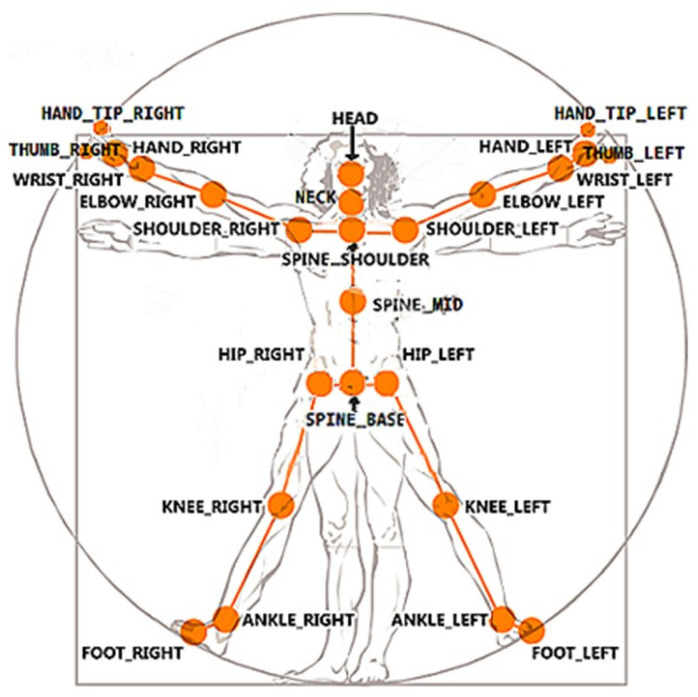
Kinect v2 25-joint skeleton system (https://lisajamhoury.medium.com/understanding-kinect-v2-joints-and-coordinate-system-4f4b90b9df16, accessed on 25 February 2025).

Kinectron with Kinect for Windows supports broadcasting one feed (frame) or multiple feeds (frames) simultaneously. Multiframe mode broadcasts several frames simultaneously in a single feed. Of the available frames, “Color” and “Skeleton” frames were tracked (Figure 3). “Color” returned a WebP file from the color camera. “Skeleton (Tracked Bodies)” provided 3D positions (X, Y, and Z coordinates) and tracking of 25 body points. These body points were the head; neck; spine at shoulder level, mid-spine level, and spine-base level; and right and left shoulder, elbow, wrist, hand, thumb, hand tip, hip, knee, ankle, and foot (Figure 4). Kinect’s coordinate system is defined as follows: X is the mediolateral axis, Y is the vertical axis, and Z is the posteroanterior axis. The system’s origin (x = 0, y = 0, z = 0) is located at the center of the Kinect’s infrared sensor.

“Skeleton” body data were sent over the peer connection as .json files, including a timestamp. Based on the .json file, a script was written to create .csv files (a separate file for each bodyIndex map) and generate a final Microsoft Excel document with several worksheets from these .csv files (each bodyIndex map on a separate sheet). After Euclidean matching and DTW (data time warping) of three sensors, due to inconsistencies, control of the final output regarding latency, different processing speeds, missing or erroneous data, etc., was managed through manual identification and alignment of selected significant strides. This process integrated technical inspection of raw data supported by video analysis and control by a clinician. The most representative gait cycle was selected for each participant. The selection process had two phases: (1) visual exclusion of less coherent trials and (2) expert decision on the most representative trials corresponding to the natural gait of participants. Selected spatiotemporal and kinematic parameters were analyzed for the chosen gait cycle. Kinect v2 definitions of these parameters are presented in Table 1. Marker locations and vectors used to calculate these parameters were extracted by processing the recorded “Skeleton“ motion sequences.

Three Kinect v2 sensors were used; S1 was the main sensor and S2 and S3 were used for the control of the measurement (Figure 1). For the first randomly selected participant, data from each Kinect sensor were separately analyzed. As the results for gait parameters acquired from control sensors S2 and S3 were within the Standard Error of the Mean, further analyses were based on the data from sensor S1. The relative positions of the sensors were in line with the findings of Gruić et al. [38] (who tested the sensor’s focal point—‘soft spot’), and the orientations were established after pilot measurements, which included the setups and methods found in previous studies by Ryselis et al. [39], Córdova-Esparza et al. [40], Guffanti et al. [41], etc.

### 2.5. Statistical Analysis

Statistical analysis was performed using Statistica (TIBCO Statistica v14.0.0) software package for Windows (Cloud Software Group, Santa Clara, CA, USA). The Kolmogorov–Smirnov test was used to confirm normal data distribution. Based on this, the data were represented as the mean ± standard deviation (SD) for continuous variables and n (%) for categorical variables. Parameters were defined as the average results of all participants in each group. As every parameter represented normal distribution, independent samples *t*-tests were used to compare AS and HC groups. A *p*-value < 0.05 was considered statistically significant. The effect size was determined by calculating the mean difference between the groups and dividing the result by the pooled standard deviation.

*Cohen*’*s d* = (*M*_2_ − *M*_1_)/*SD_pooled_*

*SD_pooled_* = √ ((*SD*_1_^2^ + *SD*_2_^2^)/2)

This was interpreted according to Cohen’s conventions (*d* = 0.20, small; *d* = 0.50, medium; and *d* = 0.80, large).

Correlation analysis and analysis of covariance (ANCOVA) were performed.

The results of ANCOVA were confirmed by a post hoc Tukey’s test.

## 3. Results

### 3.1. Demographic and Anthropometric Characteristics

In the AS group, 10/12 (83%) participants were male, and in the HC group, 9/12 (75%) participants were male. The mean duration of the disease in the AS group was 14.42 years (minimum 2–maximum 30).

The demographic and anthropometric characteristics of both groups and their comparison are presented in Table 2.

There was a statistically significant difference in the age between the groups, with the AS group being older than the HC group (AS: 47.42 [SD 9.39] vs. HC: 38.67 [SD 6.64] years; *p* < 0.05). This was accompanied by a Cohen’s d effect size of 1.07, suggesting a large difference between the groups for this variable. The statistical power for the differences in age at *p* = 0.01 was 0.65, and at *p* = 0.05 it was 0.87.

No significant differences were found between the two groups in their height, weight, or length and width of their feet.

### 3.2. Gait Parameters

The gait parameters and comparison between the AS and HC groups are reported in Table 3.

The dynamic pedobarographic peak forces were significantly higher in the midfoot area in the AS group compared to the healthy controls (AS: 235.36 [SD 111.03] vs. HCs: 149.94 [SD 76.84] N/cm^2^; *p* < 0.05), with a large Cohen’s d effect size of 0.90. The statistical power for the differences in the midfoot at *p* = 0.01 was 0.40 and at *p* = 0.05 it was 0.69. There was no significant difference in the dynamic maximum force between the groups.

The static measurements showed a significantly lower relative pressure distribution in Q2 in the AS group compared to the healthy control group (AS: 22.47 [SD 1.67] vs. HC: 25.59 [SD 3.08] %; *p* = 0.01), with a large Cohen’s d effect size of 1.26. The statistical power for the differences in Q2 at *p* = 0.01 was 0.72 and at *p* = 0.05 it was 0.91. A significantly higher relative pressure distribution was found in Q4 in the AS group compared to the HC group (AS: 27.17 [SD 4.00] vs. HC: 24.04 [SD 3.38] %; *p* = 0.05), with a large Cohen’s d effect size of 0.85. The statistical power for the differences in Q4 at *p* = 0.01 was 0.35 and at *p* = 0.05 it was is 0.64.

The AS group had a significantly shorter stride length than the healthy controls (AS: 1.13 [SD 0.12] vs. HCs: 1.27 [SD 0.11] m; *p* = 0.01), with a large Cohen’s d effect size of 1.22. The statistical power for the differences in stride length at *p* = 0.01 was 0.68 and at *p* = 0.05 it was 0.89.

No significant differences were found between the groups in their kinematic parameters: hip flexion/extension and adduction/abduction, knee flexion/extension, and ankle plantar flexion/dorsal flexion angles. Although not statistically significant, a pattern of smaller values in every measured joint angle in the right leg compared to the left leg in both groups was observed.

**Figure 5:**
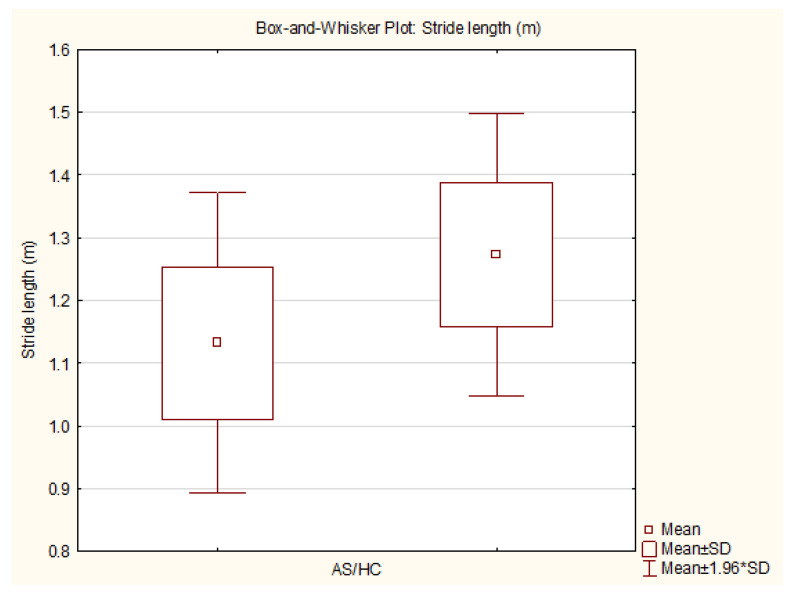
Box-and-whisker plot—stride length AS/HC (generated by TIBCO Statistica v14.0.0).

**Figure 6:**
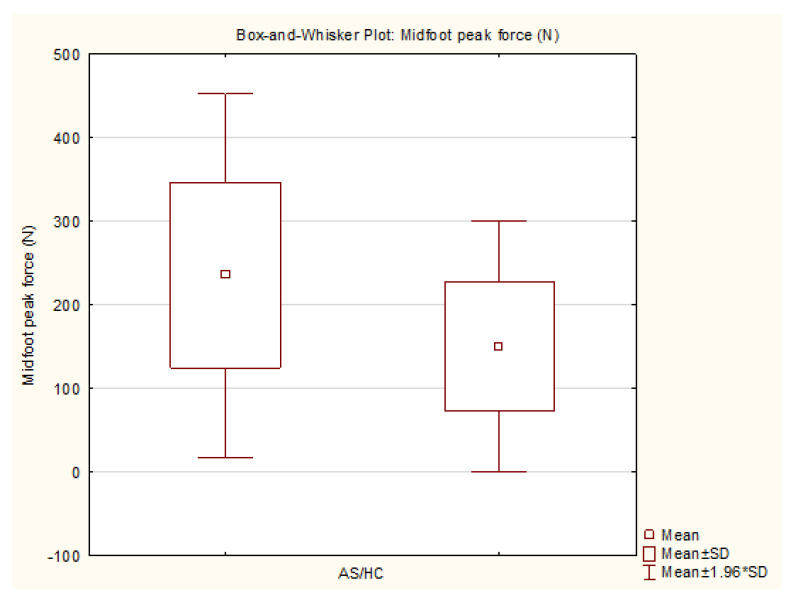
Box-and-whisker plot—midfoot peak force AS/HC (generated by TIBCO Statistica v14.0.0).

**Figure 7:**
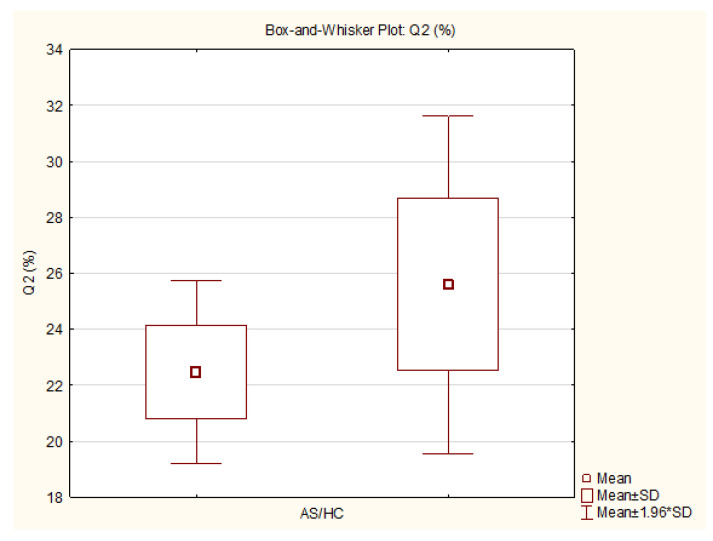
Box-and-whisker plot—front-right quadrant (Q2) AS/HC (generated by TIBCO Statistica v14.0.0).

**Figure 8:**
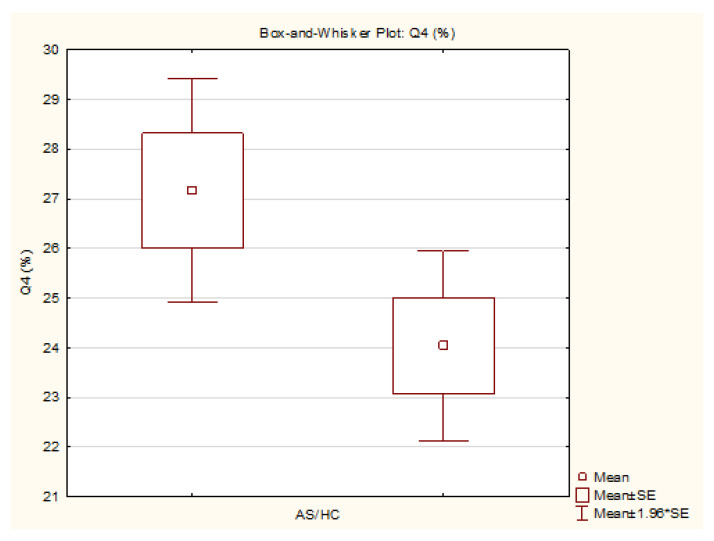
Box-and-whisker plot—rear-right quadrant (Q4) AS/HC (generated by TIBCO Statistica v14.0.0).

The variables with statistically significant differences are represented as box-and-whisker plots (Figure 5, Figure 6, Figure 7 and Figure 8).

### 3.3. Analysis of Covariance

The ANCOVA revealed age as a covariate with the dependent variable stride length (F = 12.1862; *p* = 0.002179). Therefore, the differences between the AS and HC groups, although statistically significant, should be taken with caution due to the statistically significant interference between the age-related and disease-related influences.

The results of the ANCOVA regarding the age covariations with the midfoot peak pressure (F= 0.021593; *p* = 0.884577) showed a limited influence on the statistical significance of the differences between the AS and HC groups (F = 3.760174; *p* = 0.066041). Although the differences were not statistically significant with the excluded covariates, with a *p*-value near significance (*p* = 0.066041), the results should be taken as (conditionally) valid.

The ANCOVA did not reveal age as a covariate with Q2 (F = 0.38972; *p* = 0.539172), so the differences between the AS and HC groups remained statistically significant.

The ANCOVA revealed that age as a covariate with Q4 (F = 0.03; *p* = 0.87) has a very limited or no relevance for understanding any potential differences.

All four results were confirmed by a post hoc Tukey’s test. A comprehensive analysis with the ANCOVA and Tukey’s tests can be found in the Supplementary Materials (Tables S4–S11).

### 3.4. Correlation Analysis

The correlations between the kinetic and kinematic parameters support previous findings that the heel peak force has a significant positive correlation with the stride length in the HC group (r = 0.58; *p* = 0.05), and is not statistically significant in the AS group. Patients with AS have a lower ground reaction force during a ‘heel strike’ phase and compensate for this by reducing their stride length, compared to the ‘less cautious’ first contact of HCs during a heel strike. The heel peak force (N) in the AS group—477.31 [SD 97.02]—was similar to that of the HCs—475.87 [SD 77.03]—but smaller, relatively, to the peak force (Max F) (N) in the AS group—1009.56 [SD 176.05] and in the HCs—911.36 [SD 121.23], respectively. Furthermore, the forefoot peak force in a healthy gait pattern is highly correlated with the peak force (significantly positive in both groups). An interesting, significant positive correlation occurred in the AS group between the heel peak force and peak force (Max F) (r = 0.61; *p* = 0.05), revealing an opposite mechanism. A significant negative correlation between the forefoot peak force and Q4 in the AS group was a single relation between the static and dynamic pedobarographic data—supporting the unilateral compensations of the AS group. A negative relation between the frontal quadrants (Q1, Q2) with the rear ones (Q3, Q4) in normal balance was found in the HC group, but not in the AS group. There was a significant negative correlation between Q3 and Q4 (r = −0.58; *p* = 0.05).

**Figure 9:**
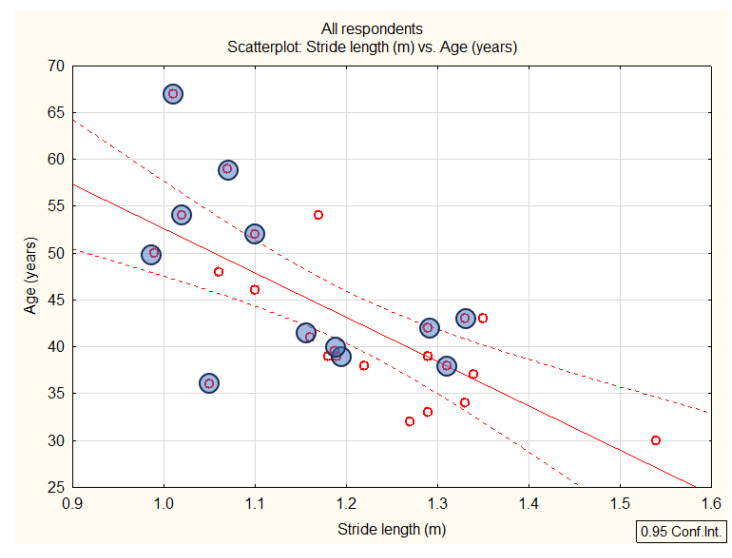
Scatterplot—stride length (m) vs. age (years) (red—all respondents; blue—AS patients).

The relationship between age and stride length is a systemic one. Older age was correlated with a shorter stride in both groups (correlation in the AS group was r = −0.60 and *p* < 0.05; and in the HC group r = −0.63 and *p* < 0.05). The age–stride length correlation scatterplot is presented in Figure 9. None of the correlations of age with the other variables were statistically significant, except for the heel peak force in the AS group, presumably due to the supra-summation of the age-related and disease-related changes that decrease movement control (for future research; the effect size was insufficient with low statistical power). In our study, the average age of the AS group was 47, and for the HCs it was 39. According to the study by Rossler et al. [42], stride length in women begins to shorten only after the age of 60 and in men after the age of 70, thereby partially negating the importance of age as the dominant reason for the shortening stride-related covariates in this sample of participants. Moreover, the influence of disease in the AS group lasted longer on average (14.42 years) compared to the average difference in age between the AS and HC groups (8.75 years). The analysis of covariance can partially explain the concurrence of age-related influences and disease-related differences, but not conclusively in total.

The comprehensive results of the correlation analysis for all the variables can be found in the Supplementary Materials (Tables S1–S3).

## 4. Discussion

### 4.1. Findings

There have been five previous studies that evaluated spatiotemporal and kinematic gait parameters [23,24,25,26,27], and only one that used dynamic pedobarography [29] to assess the gait of patients with AS. However, no previous study has combined these methods in one comprehensive research study. In our study, we used this approach to assess the gait of AS patients.

The mean age of our AS group was significantly higher than that of the HC group, which could have impacted the gait parameters [43,44]. The mean age of the AS and HC groups in our study was comparable to those reported in previous studies (the mean age of the AS patients was between 40.2 and 49.4 years, and the mean age of the healthy controls was between 39.5 and 55.8 years) [23,24,25,26]. The lack of significant differences between the groups in their height, weight, or length and width of their feet reflects the morphological comparability of the participants.

In the static pedobarographic measurements, we found a significantly lower relative pressure distribution in the front-right quadrant (Q2) and a significantly higher relative pressure distribution in the rear-right quadrant (Q4) in the AS group compared to the healthy control group. Vergara et al. [45] argued that standing postural control is altered among AS patients, and our results support this, although just indirectly, through the pedobarographic measurement. We hypothesized that the forward and downward shift of the center of mass in AS patients, as described by Bot et al. [8], would result in increased forefoot and midfoot plantar pressures. A study by Mesci et al. [28] described the displacement of plantar pressure distribution in AS patients as toward the heels, a result that is in line with our work. Other studies that have used static pedobarography for postural assessment [29,30] have not found a significant difference in the pressure distribution between patients with AS and healthy controls. The results of our research and these studies [28,29,30] could point to the successful postural compensations of AS patients with their lower limbs, using hip extension, knee flexion, and ankle plantar flexion, as described in previous studies [8,29].

Our results show a significantly increased dynamic midfoot pressure in the AS patients compared to the healthy controls, which is in partial agreement with Aydin et al. [29], who found increased dynamic forefoot and midfoot pressures in AS patients. This may indicate a loss of the proposed static postural compensations during dynamic activity (gait), which would result in an altered loading pattern due to the displaced center of mass. This could lead to a dynamic balance impairment. Relatively little emphasis has so far been placed on balance exercises in the design of rehabilitation programs for AS patients [46]. So, as a therapeutic intervention, we recommend adding dynamic balance exercises to AS patients’ rehabilitation programs.

Regarding the spatiotemporal parameters, our results show a significantly shorter stride length in the AS group compared to the healthy control group. This result is in agreement with the decrease in stride length of AS patients reported by Zebouni et al. [23] and Zhang et al. [26], and also by Soulard et al. in patients with axial spondyloarthritis [47]. However, our result is not in line with the studies by Del Din et al. [25] and Mangone et al. [24], which did not report any significant differences in stride length between AS patients and healthy controls. A shorter stride length may indicate that AS patients use a more cautious gait, as mentioned in previous studies [23,25].

No significant differences between the groups were revealed in our study with respect to their hip flexion/extension, hip adduction/abduction, knee flexion/extension, or ankle plantar flexion/dorsal flexion angles. These results are not in line with previous studies. Three studies have reported a decrease in hip flexion [23,25,26], and one study reported a decrease in hip extension in AS patients compared to healthy controls [24]. It should be noted that the AS patients in a study by Zhang et al. [26] had radiographically verified hip involvement, which could explain a decrease in hip flexion. However, as that was not the case in the other studies, this result can only be partially attributed to hip involvement. Two studies have reported increased hip abduction in AS patients [25,26]. Analyses of knee angles have shown inconclusive results: Del Din et al. [25] found decreased knee flexion in AS patients, while Zhang et al. [26] reported that patients with AS had increased knee flexion during the stance phase of the gait cycle and decreased knee flexion during the swing phase. No significant difference in the knee angle between AS patients and healthy controls was revealed by Zebouni et al. [23]. Regarding ankle movements in the sagittal plane, Zhang et al. [26] found a decreased plantar flexion in AS patients, while Del Din et al. [25] found a decreased dorsal flexion at initial contact. An explanation for the inability of our study to reveal any significant differences in the kinematic parameters of the AS patients could be that our small sample size was insufficient to show minor differences in the angles of the evaluated joints.

Considering that the AS patients evaluated in our study did not have any significant pain at the time of the gait analysis, the observed differences in the gait parameters do not represent antalgic adjustments.

Overall, the data provided in this study can be utilized for the precise modification of rehabilitation interventions aimed at improving the gait patterns of AS patients. Instrumentation analogous to that used in this study has the potential for everyday clinical application, as an affordable and simple tool that could provide a valuable upgrade to the standard observational gait analysis.

### 4.2. Study Limitations and Future Research

Our study has some limitations that should be noted. First, the sample size was small, which could affect the reliability of the results. Second, the AS group was significantly older than the control group, which could have impacted the gait parameters. Third, foot deformations (for example, flat foot) or arthritis/enthesitis in the foot region were not considered as exclusion criteria, and foot pathology was not analyzed in this study by any means other than the gait analysis. It should be noted that in this study, only one stride of each participant was analyzed, which could have skewed the averaged data. For the spatiotemporal and kinematic evaluation of the gait, we used a Kinect v2 markerless motion capture system. Although several studies have confirmed Kinect v2 as a valid and acceptable tool for gait analysis, there are limitations in the analysis of ankle movements using this system [15,16,17,18]. Marker-based motion capture systems are still considered the gold standard for gait analysis. However, while they provide the most accurate measurement of gait, they are considerably more expensive, can only be used in a controlled laboratory setting, and require skilled personnel for setup and data collection. One of the objectives of our study was to test a simple and affordable gait analysis system that could easily be used in any clinical setting. Future applications of similar multi-sensor system setups (more recent RGB-D systems, such as Azure Kinect, Stereolabs ZED, Intel RealSense, and others) should include a comparison of the measurements against a gold-standard system, such as Vicon, to assess the precision and reliability (well presented in, e.g., Guffanti et al. [41]). Although Microsoft Kinect v2 is no longer manufactured, it is still a regular asset of many laboratories, and the SDK (software development kit) and support are still accessible. There is still a community producing scientific contributions utilizing Kinect v2. Regarding the evaluated gait parameters, our measurements were limited to the main spatiotemporal and kinematic parameters of the lower limbs. Shoulder, trunk, and pelvis kinematics should be included in future studies to provide a broader perspective on postural and gait impairment in AS patients. Electromyography could also be added to study protocols as a valuable method for the direct assessment of muscle strength and its effect on gait. As this was a cross-sectional study, future longitudinal research could focus on the effects of pharmacotherapy and physical therapy on gait pattern in AS patients.

## 5. Conclusions

To our knowledge, this is the first study to use a markerless motion capture system for gait analysis in patients with AS. It is also the first study of this population to combine a kinematic and pedobarographic gait analysis. This study provides results that support the thesis that postural control and gait patterns are altered in patients with AS. We found significant differences between the AS patients and the healthy controls in the static and dynamic pedobarographic measurements and stride length. We did not find a significant difference in the kinematic lower-limb joint angles. Considering that the AS patients were pain-free at the time of the analysis, those differences represent a feature of AS and not antalgic adjustments. Rehabilitation programs for AS patients should include balance and gait exercises tailored according to the objectives and quantifiable results obtained by an instrumented gait analysis.

## Figures and Tables

**Table 1:** Definitions of parameters for Kinect v2 system.

Parameter	Kinect v2
Stride length (m)	Distance between the right ankle markers in the Z-axis for two consecutive right foot strikes. Foot strike was identified as the moment when the distance between the ankle marker and spine base marker reached the maximum. The ankle marker was used due to its better tracking performance than the foot marker.
Hip joint flexion/extension angle (°)	Angle between the “hip-knee vector” and the “Y axis” in the Y-Z plane.
Hip joint adduction/abduction angle (°)	Angle between the “hip-knee vector” and the “Y axis” in the X-Y plane.
Knee joint flexion/extension angle (°)	Angle between the “knee-hip vector” and the “knee-ankle vector” in the Y-Z plane.
Ankle joint dorsiflexion/plantarflexion angle (°)	Angle between the “ankle-knee vector” and the “ankle-foot vector” in the Y-Z plane.

**Table 2:** Demographic and anthropometric characteristics of ankylosing spondylitis (AS) group (*n* = 12) and healthy control (HC) group (*n* = 12).

	AS	HC	AS vs. HC	
Variable	Mean ± SD	Mean ± SD	*p*-Value (*t*-Test)	Effect Size
Age (years)	47.42 ± 9.39	38.67 ± 6.64	0.02 *	1.07
Height (cm)	173.33 ± 5.69	178.50 ± 9.08	0.11	0.67
Weight (kg)	87.83 ± 15.57	77.19 ± 12.47	0.08	0.75
Right foot length (cm)	28.77 ± 1.60	28.78 ± 1.94	0.48	0.01
Right foot width (cm)	10.78 ± 0.44	10.62 ± 0.61	0.48	0.30
Left foot length (cm)	29.25 ± 1.37	28.63 ± 2.02	0.39	0.36
Left foot width (cm)	10.80 ± 0.58	10.62 ± 0.63	0.48	0.30

* Statistically significant.

**Table 3:** Pedobarographic, spatiotemporal, and kinematic gait parameters of AS (*n* = 12) and HC (*n* = 12) groups.

	AS	HC	AS vs. HC	
Variable	Mean ± SD	Mean ± SD	*p*-Value (*t*-Test)	Effect Size
Forefoot peak force (N)	886.64 ± 148.66	818.24 ± 119.64	0.23	0.51
Midfoot peak force (N)	235.35 ± 111.03	149.94 ± 76.84	0.04 *	0.90
Heel peak force (N)	477.31 ± 97.02	475.87 ± 77.03	0.97	0.02
Peak force (N)	1009.56 ± 176.05	911.36 ± 121.23	0.13	0.65
Q1 (%)	23.16 ± 4.56	25.56 ± 3.43	0.16	0,60
Q2 (%)	22.47 ± 1.67	25.59 ± 3.08	0.01 *	1.26
Q3 (%)	27.22 ± 4.66	24.81 ± 3.86	0.18	0.56
Q4 (%)	27.17 ± 4.00	24.04 ± 3.38	0.05 *	0.85
Stride length (m)	1.13 ± 0.12	1.27 ± 0.11	0.01 *	1.22
Right hip flexion/extension (°)	18.30 ± 5.97	18.71 ± 3.99	0.84	0.08
Left hip flexion/extension (°)	24.40 ± 4.90	24.46 ± 3.60	0.97	0.01
Right hip adduction/abduction (°)	20.55 ± 3.56	22.72 ± 7.41	0.37	0.37
Left hip adduction/abduction (°)	23.33 ± 8.26	23.28 ± 7.49	0.99	0.01
Right knee flexion/extension (°)	37.92 ± 9.45	41.02 ± 8.71	0.41	0.34
Left knee flexion/extension (°)	49.96 ± 13.51	49.05 ± 14.16	0.87	0.07
Right ankle dorsiflexion/plantarflexion (°)	55.80 ± 15.61	51.14 ± 14.89	0.46	0.31
Left ankle dorsiflexion/plantarflexion (°)	59.72 ± 16.02	58.52 ± 11.16	0.83	0.09

* Statistically significant.

## Data Availability

Part of the dataset is available on request from the authors (the data are part of an ongoing project).

## Supplementary Materials

The following supporting information can be downloaded at: https://www.mdpi.com/article/10.3390/s25061797/s1.
