# Supplementary material for: Optomechanical Analysis of Gait in Patients with Ankylosing Spondylitis
Source: Sensors (Basel). 2025 Mar 14;25(6):1797. doi: 10.3390/s25061797 (PMC11945937; doi:10.3390/s25061797)
Supplement: Supplementary file 1 [file sensors-25-01797-s001.zip › sensors-3492596-supplementary.pdf]

Table S1. AS+HC correlations; marked correlations are significant at $p < 0.05$, N=24.

Variable	Mean	Standard deviation	Stride length	Left ankle dorsiflexion/plantarflexion	Right ankle dorsiflexion/plantarflexion	Left knee flexion/extension	Right knee flexion/extension	Left hip flexion/extension	Right hip flexion/extension	Left hip adduction/abduction	Right hip adduction/abduction	Forefoot peak force	Midfoot peak force	Heel peak force	Max F	Q1	Q2	Q3	Q4	Age	Height	Weight
Stride length	1.20	0.14	1.00	-0.09	0.11	-0.08	0.08	0.19	0.06	-0.06	-0.02	-0.19	-0.34	0.10	-0.30	0.00	0.43	-0.18	0,14	-0.71	0.49	-0.30
Left ankle dorsiflexion/plantarflexion	59.12	13.51	-0.09	1.00	0.32	0.23	0.32	0.24	0.57	0.38	0.08	-0.44	-0.04	-0.35	-0.51	-0.00	0.01	-0.04	0,23	0.05	-0.24	-0.45
Right ankle dorsiflexion/plantarflexion	53.47	15.11	0.11	0.32	1.00	0.23	0.21	0.17	0.30	0.25	-0.11	-0.04	-0.09	0.23	-0.04	-0.10	-0.00	0.02	0,19	0.31	-0.25	-0.09
Left knee flexion/extension	49.51	13.54	-0.08	0.23	0.23	1.00	0.53	0.25	0.39	0.30	0.36	0.07	-0.16	0.10	-0.03	-0.27	0.21	-0.01	0,24	0.32	-0.13	-0.17
Right knee flexion/extension	39.47	9.03	0.08	0.32	0.21	0.53	1.00	0.13	0.34	0.30	0.54	-0.22	0.00	0.11	-0.25	-0.15	0.27	-0.24	0,35	0.10	-0.02	-0.30
Left hip flexion/extension	24.43	4.20	0.19	0.24	0.17	0.25	0.13	1.00	0.42	0.00	-0.01	0.05	-0.11	-0.09	-0.09	0.09	0.13	-0.34	0,11	0.04	-0.05	-0.27
Right hip flexion/extension	18.51	4.97	0.06	0.57	0.30	0.39	0.34	0.42	1.00	0.13	0.32	-0.15	-0.14	-0.24	-0.36	-0.16	-0.08	-0.01	-0,14	0.08	-0.28	-0.41
Left hip adduction/abduction	23.31	7.71	-0.06	0.38	0.25	0.30	0.30	0.00	0.13	1.00	0.15	-0.18	-0.38	0.04	-0.15	-0.18	-0.04	-0.12	0,18	0.27	-0.39	-0.23
Right hip adduction/abduction	21.64	5.79	-0.02	0.08	-0.11	0.36	0.54	-0.01	0.32	0.15	1.00	0.11	0.04	0.07	0.05	-0.30	-0.05	0.22	-0,18	-0.00	-0.18	0.00
Forefoot peak force	852.44	136.51	-0.19	-0.44	-0.04	0.07	-0.22	0.05	-0.15	-0.18	0.11	1.00	0.07	0.45	0.93	0.01	-0.27	0.30	-0,10	0.44	0.12	0.75
Midfoot peak force	192.65	103.07	-0.34	-0.04	-0.09	-0.16	0.00	-0.11	-0.14	-0.38	0.04	0.07	1.00	-0.19	0.13	-0.08	-0.37	0.16	-0,56	0.18	-0.05	0.25
Heel peak force	476.58	85.67	0.10	-0.35	0.23	0.10	0.11	-0.09	-0.24	0.04	0.07	0.45	-0.19	1.00	0.57	-0.06	0.11	0.14	-0,37	0.33	0.40	0.66
Peak force (Max F)	960.46	156.10	-0.30	-0.51	-0.04	-0.03	-0.25	-0.09	-0.36	-0.15	0.05	0.93	0.13	0.57	1.00	-0.04	-0.23	0.28	-0,13	0.49	0.13	0.85
Q1	24.36	4.13	0.00	-0.00	-0.10	-0.27	-0.15	0.09	-0.16	-0.18	-0.30	0.01	-0.08	-0.06	-0.04	1.00	0.20	-0.57	1,00	-0.09	0.30	-0.01
Q2	24.03	2.90	0.43	0.01	-0.00	0.21	0.27	0.13	-0.08	-0.04	-0.05	-0.27	-0.37	0.11	-0.23	0.20	1.00	-0.52	0,17	-0.37	0.43	-0.35
Q3	26.01	4.36	-0.18	-0.04	0.02	-0.01	-0.24	-0.34	-0.01	-0.12	0.22	0.30	0.16	0.14	0.28	-0.57	-0.52	1.00	-0,36	0.18	-0.25	0.38
Q4	25,60	3,96	-0,12	0,05	0,10	0,14	0,23	0,19	0,24	0,35	0,11	-0,14	0,18	-0,18	-0,10	-0,56	-0,37	-0,13	-0,15	0,17	-0,36	-0,15
Age	43.04	9.12	-0.71	0.05	0.31	0.32	0.10	0.04	0.08	0.27	-0.00	0.44	0.18	0.33	0.49	-0.09	-0.37	0.18	0,14	1.00	-0.40	0.41
Height	175.92	7.87	0.49	-0.24	-0.25	-0.13	-0.02	-0.05	-0.28	-0.39	-0.18	0.12	-0.05	0.40	0.13	0.30	0.43	-0.25	0,23	-0.40	1.00	0.24
Weight	82.51	14.83	-0.30	-0.45	-0.09	-0.17	-0.30	-0.27	-0.41	-0.23	0.00	0.75	0.25	0.66	0.85	-0.01	-0.35	0.38	0,19	0.41	0.24	1.00

Table S2. AS correlations; marked correlations are significant at $p < 0.05$, N=12.

Variable	Mean	Standard deviation	Stride length	Left ankle dorsiflexion/plantarflexion	Right ankle dorsiflexion/plantarflexion	Left knee flexion/extension	Right knee flexion/extension	Left hip flexion/extension	Right hip flexion/extension	Left hip adduction/abduction	Right hip adduction/abduction	Forefoot peak force	Midfoot peak force	Heel peak force	Max F	Q1	Q2	Q3	Q4	Age	Height	Weight
Stride length	1.13	0.12	1.00	0.22	0.28	0.09	0.18	0.30	0.27	-0.23	-0.40	-0.15	-0.08	-0.22	-0.39	-0.37	0.17	0.25	0.05	**-0.60**	0.38	-0.44
Left ankle dorsiflexion/plantarflexion	59.71	16.02	0.22	1.00	0.34	0.20	0.38	0.53	**0.71**	0.38	0.19	**-0.68**	-0.12	-0.27	**-0.77**	-0.01	0.03	-0.16	0.20	-0.09	-0.19	-0.57
Right ankle dorsiflexion/plantarflexion	55.80	15.61	0.28	0.34	1.00	0.55	0.53	0.44	**0.58**	0.12	0.28	0.22	-0.17	0.55	0.07	-0.11	0.13	0.22	-0.18	0.50	-0.16	0.07
Left knee flexion/extension	49.96	13.51	0.09	0.20	0.55	1.00	**0.71**	0.39	**0.58**	0.58	0.27	0.10	-0.39	0.35	-0.05	-0.06	0.31	-0.36	0.35	0.49	0.12	-0.22
Right knee flexion/extension	37.92	9.45	0.18	0.38	0.53	**0.71**	1.00	0.42	0.54	0.42	0.33	-0.29	0.04	0.36	-0.31	-0.21	0.35	-0.32	0.47	0.38	0.17	-0.30
Left hip flexion/extension	24.40	4.90	0.30	0.53	0.44	0.39	0.42	1.00	0.55	0.21	-0.22	-0.15	-0.26	-0.24	-0.29	0.12	0.46	-0.49	0.25	0.09	-0.18	**-0.62**
Right hip flexion/extension	18.30	5.97	0.27	**0.71**	**0.58**	**0.58**	0.54	0.55	1.00	0.29	0.22	-0.39	-0.38	-0.04	-0.57	-0.18	0.02	-0.22	0.46	0.12	-0.32	-0.55
Left hip adduction/abduction	23.33	8.25	-0.23	0.38	0.12	0.58	0.42	0.21	0.29	1.00	0.12	-0.16	-0.41	0.17	-0.14	-0.01	0.46	-0.42	0.30	0.44	-0.21	-0.21
Right hip adduction/abduction	20.55	3.55	-0.40	0.19	0.28	0.27	0.33	-0.22	0.22	0.12	1.00	-0.11	0.08	0.54	0.01	-0.19	-0.21	0.30	-0.05	0.47	0.09	0.19
Forefoot peak force	886.64	148.66	-0.15	**-0.68**	0.22	0.10	-0.29	-0.15	-0.39	-0.16	-0.11	1.00	-0.33	0.55	**0.93**	0.26	-0.07	0.26	**-0.58**	0.45	0.07	**0.69**
Midfoot peak force	235.35	111.03	-0.08	-0.12	-0.17	-0.39	0.04	-0.26	-0.38	-0.41	0.08	-0.33	1.00	-0.15	-0.17	0.04	-0.09	0.05	-0.06	-0.17	0.11	0.07
Heel peak force	477.31	97.02	-0.22	-0.27	0.55	0.35	0.36	-0.24	-0.04	0.17	0.54	0.55	-0.15	1.00	**0.61**	-0.14	-0.00	0.42	-0.33	**0.74**	0.09	**0.65**
Peak force (Max F)	1009.56	176.05	-0.39	**-0.77**	0.07	-0.05	-0.31	-0.29	-0.57	-0.14	0.01	**0.93**	-0.17	**0.61**	1.00	0.17	0.01	0.27	-0.52	0.52	0.02	**0.80**
Q1	23.16	4.56	-0.37	-0.01	-0.11	-0.06	-0.21	0.12	-0.18	-0.01	-0.19	0.26	0.04	-0.14	0.17	1.00	-0.48	-0.43	-0.44	0.28	-0.11	0.20
Q2	22.47	1.67	0.17	0.03	0.13	0.31	0.35	0.46	0.02	0.46	-0.21	-0.07	-0.09	-0.00	0.01	-0.48	1.00	-0.25	0.42	0.06	-0.05	-0.36
Q3	27.22	4.66	0.25	-0.16	0.22	-0.36	-0.32	-0.49	-0.22	-0.42	0.30	0.26	0.05	0.42	0.27	-0.43	-0.25	1.00	**-0.58**	-0.15	0.20	0.48
Q4	27.17	4.00	0.05	0.20	-0.18	0.35	0.47	0.25	0.46	0.30	-0.05	**-0.58**	-0.06	-0.33	-0.52	-0.44	0.42	**-0.58**	1.00	-0.17	-0.09	**-0.63**
Age	47.42	9.39	**-0.60**	-0.09	0.50	0.49	0.38	0.09	0.12	0.44	0.47	0.45	-0.17	**0.74**	0.52	0.28	0.06	-0.15	-0.17	1.00	-0.33	0.44
Height	173.33	5.69	0.38	-0.19	-0.16	0.12	0.17	-0.18	-0.32	-0.21	0.09	0.07	0.11	0.09	0.02	-0.11	-0.05	0.20	-0.09	-0.33	1.00	0.07
Weight	87.83	15.57	-0.44	-0.57	0.07	-0.22	-0.30	**-0.62**	-0.55	-0.21	0.19	**0.69**	0.07	**0.65**	**0.80**	0.20	-0.36	0.48	**-0.63**	0.44	0.07	1.00

Table S3. HC correlations: marked correlations are significant at $p < 0.05$, N=12.

Variable	Mean	Standard deviation	Stride length	Left ankle dorsiflexion/plantarflexion	Right ankle dorsiflexion/plantarflexion	Left knee flexion/extension	Right knee flexion/extension	Left hip flexion/extension	Right hip flexion/extension	Left hip adduction/abduction	Right hip adduction/abduction	Forefoot peak force	Midfoot peak force	Heel peak force	Max F	Q1	Q2	Q3	Q4	Age	Height	Weight
Stride length	1.27	0.11	1.00	-0.52	0.17	-0.24	-0.26	0.11	-0.32	0.12	-0.03	0.05	-0.27	0.58	0.17	0.06	0.22	-0.41	0,21	-0.63	0.41	0.28
Left ankle dorsiflexion/plantarflexion	58.52	11.16	-0.52	1.00	0.30	0.28	0.26	-0.31	0.29	0.38	0.05	-0.08	0.05	-0.51	-0.10	0.06	0.05	0.13	-0,25	0.29	-0.33	-0.38
Right ankle dorsiflexion/plantarflexion	51.14	14.89	0.17	0.30	1.00	-0.08	-0.09	-0.21	-0.10	0.40	-0.28	-0.49	-0.20	-0.18	-0.35	0.01	0.10	-0.35	0,31	-0.06	-0.25	-0.47
Left knee flexion/extension	49.05	14.16	-0.24	0.28	-0.08	1.00	0.37	0.09	0.15	0.02	0.45	0.02	0.06	-0.20	-0.03	-0.54	0.27	0.36	-0,10	0.18	-0.28	-0.16
Right knee flexion/extension	41.02	8.71	-0.26	0.26	-0.09	0.37	1.00	-0.30	0.03	0.16	0.69	-0.04	0.16	-0.22	-0.07	-0.24	0.15	-0.05	0,16	-0.01	-0.26	-0.19
Left hip flexion/extension	24.46	3.60	0.11	-0.31	-0.21	0.09	-0.30	1.00	0.15	-0.31	0.12	0.41	0.16	0.16	0.30	0.03	-0.04	-0.12	0,13	-0.01	0.05	0.27
Right hip flexion/extension	18.71	3.99	-0.32	0.29	-0.10	0.15	0.03	0.15	1.00	-0.13	0.48	0.32	0.40	-0.61	0.08	-0.18	-0.27	0.40	-0,02	0.09	-0.37	-0.20
Left hip adduction/abduction	23.28	7.49	0.12	0.38	0.40	0.02	0.16	-0.31	-0.13	1.00	0.18	-0.22	-0.45	-0.14	-0.18	-0.45	-0.36	0.27	0,49	0.12	-0.58	-0.31
Right hip adduction/abduction	22.72	7.41	-0.03	0.05	-0.28	0.45	0.69	0.12	0.48	0.18	1.00	0.37	0.22	-0.19	0.23	-0.57	-0.18	0.33	0,38	-0.11	-0.37	0.03
Forefoot peak force	818.24	119.64	0.05	-0.08	-0.49	0.02	-0.04	0.41	0.32	-0.22	0.37	1.00	0.46	0.34	0.94	-0.19	-0.25	0.22	0,17	0.23	0.37	0.80
Midfoot peak force	149.94	76.84	-0.27	0.05	-0.20	0.06	0.16	0.16	0.40	-0.45	0.22	0.46	1.00	-0.33	0.33	0.06	-0.28	0.05	0,13	0.25	0.13	0.20
Heel peak force	475.86	77.03	0.58	-0.51	-0.18	-0.20	-0.22	0.16	-0.61	-0.14	-0.19	0.34	-0.33	1.00	0.57	0.08	0.25	-0.27	0,01	-0.28	0.73	0.78
Peak force (Max F)	911.36	121.23	0.17	-0.10	-0.35	-0.03	-0.07	0.30	0.08	-0.18	0.23	0.94	0.33	0.57	1.00	-0.15	-0.15	0.11	0,17	0.16	0.54	0.91
Q1	25.56	3.43	0.06	0.06	0.01	-0.54	-0.24	0.03	-0.18	-0.45	-0.57	-0.19	0.06	0.08	-0.15	1.00	0.43	-0.71	-0,61	-0.35	0.53	-0.03
Q2	25.59	3.08	0.22	0.05	0.10	0.27	0.15	-0.04	-0.27	-0.36	-0.18	-0.25	-0.28	0.25	-0.15	0.43	1.00	-0.64	-0,61	-0.31	0.43	-0.11
Q3	24.81	3.86	-0.41	0.13	-0.35	0.36	-0.05	-0.12	0.40	0.27	0.33	0.22	0.05	-0.27	0.11	-0.71	-0.64	1.00	0,16	0.40	-0.47	0.05
Q4	24,04	3,38	0,21	-0,25	0,31	-0,10	0,16	0,13	-0,02	0,49	0,38	0,17	0,13	0,01	0,17	-0,61	-0,61	0,16	1,00	0,19	-0,40	0,08
Age	38.67	6.64	-0.63	0.29	-0.06	0.18	-0.01	-0.01	0.09	0.12	-0.11	0.23	0.25	-0.28	0.16	-0.35	-0.31	0.40	0,19	1.00	-0.30	0.02
Height	178.50	9.08	0.41	-0.33	-0.25	-0.28	-0.26	0.05	-0.37	-0.58	-0.37	0.37	0.13	0.73	0.54	0.53	0.43	-0.47	-0,40	-0.30	1.00	0.72
Weight	77.19	12.47	0.28	-0.38	-0.47	-0.16	-0.19	0.27	-0.20	-0.31	0.03	0.80	0.20	0.78	0.91	-0.03	-0.11	0.05	0,08	0.02	0.72	1.00

Table S4. ANCOVA – Stride length.

Effect	Univariate Results for Each DV; Sigma-restricted parameterization Effective hypothesis decomposition				
	Degrees of freedom	Stride length SS	Stride length MS	Stride length F	Stride length p
Intercept	1	1.902099	1.902099	204.4211	0.000000
Age	1	0.113391	0.113391	12.1862	0.002179
AS/HC	1	0.018414	0.018414	1.9789	0.174130
Error	21	0.195401	0.009305		
Total	23	0.427796			

Table S5. Tukey's HSD test - Stride length.

Cell No.	Tukey's HSD test; variable – Stride length Approximate Probabilities for Post Hoc Tests Error: Between MSE = 0.00930, df = 21.000		
	AS/HC	{1} 1.1317	{2} 1.2725
1	AS		0.001928
2	HC	0.001928	

Table S6. ANCOVA - Midfoot peak force.

Effect	Univariate Results for Each DV; Sigma-restricted parameterization Effective hypothesis decomposition			
	Midfoot peak force SS	Midfoot peak force MS	Midfoot peak force F	Midfoot peak force p
Intercept	33140.2	33140.22	3.473626	0.076398
Age	206.0	206.01	0.021593	0.884577
AS/HC	35874.0	35874.04	3.760174	0.066041
Error	200351.0	9540.53		
Total	244327.1			

Table S7. Tukey's HSD test - Midfoot peak force.

Cell No.	Tukey's HSD test; variable – Midfoot peak force Approximate Probabilities for Post Hoc Tests Error: Between MSE = 9540.5, df = 21.000		
	AS/HC	{1} 235.35	{2} 149.94
1	AS		0.044214
2	HC	0.044214	

Table S8. ANCOVA – Q2.

Effect	Univariate Results for Each DV; Sigma-restricted parameterization Effective hypothesis decomposition			
	Q2 SS	Q2 MS	Q2 F	Q2 p
Intercept	505.7002	505.7002	80.19808	0.000000
Age	2.4574	2.4574	0.38972	0.539172
AS/HC	34.8616	34.8616	5.52864	0.028549
Error	132.4184	6.3056		
Total	193.4696			

Table S9. Tukey's HSD test – Q2.

Cell No.	Tukey's HSD test; variable Q2 Approximate Probabilities for Post Hoc Tests Error: Between MSE = 6.3056. df = 21.000		
	AS/HC	{1} 22.467	{2} 25.592
1	AS		0.006272
2	HC	0.006272	

Table S10. ANCOVA – Q4.

Effect	Univariate Results for Each DV; Sigma-restricted parameterization<br>Effective hypothesis decomposition			
	Q4<br>SS	Q4<br>MS	Q4<br>F	Q4<br>p
Intercept	1	527,0364	527,0364	36,78051
Age	1	0,4220	0,4220	0,02945
AS/HC	1	48,8726	48,8726	3,41069
Error	21	300,9139	14,3292	
Total	23	359,9296		

Table S11. Tukey's HSD test – Q4.

Cell No.	Tukey's HSD test; variable Q4<br>Approximate Probabilities for Post Hoc Tests Error: Between MSE = 14,329. df = 21.000		
	AS/HC	{1}<br>27,167	{2}<br>24,042
1	AS		0,056226
2	HC	0,056226	
